# Putting forward a model for the role of the cerebellum in cocaine-induced pavlovian memory

**DOI:** 10.3389/fnsys.2023.1154014

**Published:** 2023-06-14

**Authors:** Ignasi Melchor-Eixea, Julian Guarque-Chabrera, Aitor Sanchez-Hernandez, Patricia Ibáñez-Marín, Raúl Pastor, Marta Miquel

**Affiliations:** Área de Psicobiología, Facultat de Ciències de la Salut, Universitat Jaume I, Castellón de la Plana, Spain

**Keywords:** cerebellum, drug addiction, drug memory, cocaine, DREADDs

## Abstract

Substance Use Disorder (SUD) involves emotional, cognitive, and motivational dysfunction. Long-lasting molecular and structural changes in brain regions functionally and anatomically linked to the cerebellum, such as the prefrontal cortex, amygdala, hippocampus, basal ganglia, and ventral tegmental area, are characteristic of SUD. Direct and indirect reciprocal connectivity between the cerebellum and these brain regions can explain cerebellar roles in Pavlovian and reinforcement learning, fear memory, and executive functions. It is increasingly clear that the cerebellum modulates brain functions altered in SUD and other neuropsychiatric disorders that exhibit comorbidity with SUD. In the present manuscript, we review and discuss this evidence and present new research exploring the role of the cerebellum in cocaine-induced conditioned memory using chemogenetic tools (designer receptor exclusively activated by designer drug, DREADDs). Our preliminary data showed that inactivation of a region that includes the interposed and lateral deep cerebellar nuclei reduces the facilitating effect of a posterior vermis lesion on cocaine-induced preference conditioning. These findings support our previous research and suggest that posterior vermis damage may increase drug impact on the addiction circuitry by regulating activity in the DCN. However, they raise further questions that will also be discussed.

## Introduction

Substance Use Disorder (SUD) is a devastating mental disorder associated with brain circuit alterations that lead to emotional, motivational, and cognitive dysfunction. SUD involves long-lasting molecular and structural plasticity in brain regions anatomically and functionally related to the cerebellum, including the prefrontal cortex, amygdala, hippocampus, basal ganglia, including the nucleus accumbens (NAc), and ventral tegmental area (VTA) (Sacchetti et al., 2002; Bostan et al., 2010; Chen et al., 2014; Carta et al., 2019; Holloway et al., 2019; Rondi-Reig et al., 2022; D’Ambra et al., 2023). Although the canonical view linked the cerebellum to motor control, growing evidence points to a broader modulatory function of this region (McAfee et al., 2022). It is now evident that the cerebellum modulates brain functions impaired in SUD and other neuropsychiatric disorders that exhibit comorbidity with SUD such as cognitive flexibility, executive control, and reinforcement learning (Miquel et al., 2016). Indeed, several findings link cerebellar dysfunction to neuropsychiatric conditions such as autism (Becker and Stoodley, 2013; Wang et al., 2014; Kelly et al., 2020), obsessive-compulsive disorder (OCD) (Hartmann et al., 2016; Hazari et al., 2019; Sha et al., 2020; Qing et al., 2021), attention deficit/hyperactivity disorders (ADHD) (Arnsten, 2006; Hazari et al., 2019; Sha et al., 2020; Qing et al., 2021). Nevertheless, it has taken long for the cerebellum to emerge as a relevant region for SUD (Miquel et al., 2009, 2016, 2019; Moulton et al., 2014).

As recently recognized, reciprocal connectivity between the cerebellum and the rest of the brain is crucial to understanding cerebellar function (Galliano and De Zeeuw, 2014; McAfee et al., 2022). In particular, the cerebellum modulates activity in limbic regions (Sacchetti et al., 2007; Chen et al., 2014; Frontera et al., 2020), the basal ganglia, including the NAc (Bostan et al., 2010; Bostan and Strick, 2018; Gil-Miravet et al., 2021), and the cortical control of striatal activity (Chen et al., 2014). Moreover, results from different laboratories revealed the existence of reciprocal loops between the medial prefrontal cortex (mPFC) and the cerebellum (Chen et al., 2014; Watson et al., 2014; Gil-Miravet et al., 2021; Pisano et al., 2021). One of the candidates for mediating cerebellar modulation of the mPFC is the VTA. Transneuronal viral labeling (Watabe-Uchida et al., 2012; Carta et al., 2019) and traditional tracing agent (Gil-Miravet et al., 2021) studies showed disynaptic projections from the cerebellum to the mPFC. This area includes prelimbic (PL) and infralimbic (IL) cortices. Altogether, evidence indicates that the cerebellum not only can regulate mPFC activity through direct control over dopaminergic and non-dopaminergic neurons in the VTA (Watabe-Uchida et al., 2012; Carta et al., 2019; Gil-Miravet et al., 2021) but also through alternative pathways (Forster and Blaha, 2003; Rogers et al., 2011). Recently, we have also shown that a neurotoxic lesion in the posterior vermis dramatically facilitates learning of cocaine-induced conditioned preference and increases neuronal activity in the mPFC, NAc, and all striatal subdivisions except the ventrolateral striatum, pointing at the VTA as a plausible hotspot for a modulatory action of the cerebellum on the establishment of drug preference (Gil-Miravet et al., 2021). In the present work, we review and discuss evidence of the cerebellar contribution to the establishment and maintenance of drug-induced conditioned memory and present new research using chemogenetic tools (designer receptor exclusively activated by designer drug, DREADDs). Our goal with this new exploratory study was to obtain further evidence to propose a working model capable of explaining why posterior vermis impairment facilitates drug effects on memory and increases VTA, mPFC, and basal ganglia activity (Gil-Miravet et al., 2019; Gil-Miravet et al., 2021).

## Cerebellar response of SUD patients in drug-related cue-reactivity studies

In people with SUD, cue-action-reward associations appear to be particularly strong, with these conditioned memories becoming powerful motivational triggers for drug seeking even after protracted abstinence (Everitt and Robbins, 2005; Hyman et al., 2006). Studies of cue reactivity in SUD patients have assessed the capacity of drug-associated cues to induce conditioned emotional responses and craving. These studies have shown that the presentation of drug-related cues elicits drug craving and increases cerebellar activity (for a review, see Moulton et al., 2014; Moreno-Rius and Miquel, 2017), regardless of stimulus modality (visual, olfactory, auditive, tactile) and drug type (cocaine, alcohol, cannabis, nicotine, heroin) (Grant et al., 1996; Wang et al., 1999; Martin-Sölch et al., 2001; Schneider et al., 2001; Anderson et al., 2006; Olbrich et al., 2006; Filbey et al., 2009; Lou et al., 2012; Wei et al., 2020; Al-Khalil et al., 2021). This evidence, however, does not allow us to postulate a causal relationship between cerebellar activity and craving. Although several studies found a positive correlation between self-reports of craving and cerebellar activity (Grant et al., 1996; Risinger et al., 2005), others did not (Bonson et al., 2002). Also, similar cerebellar activity patterns have been found after exposure to cocaine cues and other arousing stimuli like anger-related scripts (Kilts et al., 2003). Furthermore, activity in the cerebellum increases when fear- (Utz et al., 2015) or food-related stimuli are presented (Tomasi et al., 2015). These findings suggest a general role of the cerebellum in processing and storing reward-predicting cues and other behaviorally relevant events more than in the generation of conscious feelings such as drug craving (Moreno-Rius and Miquel, 2017). It is important to mention, nevertheless, that cerebellar activity highly correlates with addiction severity (Shen et al., 2017; Al-Khalil et al., 2021). Given that cue-reactivity studies were all conducted under withdrawal conditions, increased activity in the cerebellum might result from an apparent mismatch between exteroceptive (drug-predicting cues and context) and the absence of expected interoceptive signals (drug effects) triggered by the cue presentation. In this case, cerebellar activity could be associated with a prediction error for the internal representation that anticipated sensory and rewarding consequences of drug effects in a given context. Accordingly, blood oxygen level dependent imaging in the dorsal caudate, mPFC and the cerebellum were greater when SUD patients were in the presence of a mismatch between mentally represented drug effects and drug taking contexts (De Pirro et al., 2018). In this study, patients with a diagnosis of heroin and cocaine dependence were instructed to create a mental imagery of drug use settings (home/outside) while imaging oneself engaging in heroin or cocaine use. Activity of the fronto-striatal-cerebellar network was larger when drug taking was mentally represented in a non-associated context for drug consumption, outside-home for heroin and the home context for cocaine (De Pirro et al., 2018). Moreover, recent animal research has shown that violations of reward expectations activate climbing fibers (Heffley and Hull, 2019) and specific subpopulations of granule cells (Wagner et al., 2017).

It has also been described that an expected drug treatment evokes a larger cerebellar response to drug administration (methylphenidate) than an unexpected treatment (Volkow et al., 2003). However, challenging the mismatch hypothesis, it has been shown that receiving placebo after expecting a drug does not activate the cerebellum (Volkow et al., 2003). These findings indicate that expectations can enhance drug effects on the cerebellum, but expectancy as a process underlying placebo response does not recruit cerebellar mechanisms.

## Conditioned preferences toward drug-related cues shape neural activity and perineuronal net expression in the cerebellum

The capacity of addictive drugs to induce Pavlovian conditioning in preclinical studies has been very well documented (Bardo and Bevins, 2000; Cunningham et al., 2006; Tzschentke, 2007; McKendrick and Graziane, 2020). The most widely used animal model of Pavlovian conditioning in drug addiction has been conditioned place preference (CPP), in which distinctive contextual cues are paired with the drug. Contextual cues can be of different sensory modalities, creating a particular spatial configuration that acquires incentive value when it is paired with the drug (CS+) (McKendrick and Graziane, 2020). The conditioned response is expressed as the preference for the drug-associated context. Distinct processes contribute to the formation of drug-induced conditioned preferences including Pavlovian learning, motivation, and reward (McKendrick and Graziane, 2020). Animals showing CPP present goal-directed behavior toward drug-associated contexts (CS+), therefore expressing a conditioned preference response that involves more than merely retrieving a Pavlovian memory.

Our rodent studies on cocaine-induced CPP consistently evidenced increased neural activity (cFos) in granule cells of the vermis (Carbo-Gas et al., 2014a,b, 2017). We also found a robust enhanced expression of perineuronal nets (PNNs) around Golgi interneurons in the same cerebellar region (Carbo-Gas et al., 2017). PNNs are extracellular matrix structures expressed by the cell-body and proximal neurites of some neuronal subpopulations that generate restrictive conditions for synaptic remodeling (Grimpe and Silver, 2002; Fawcett et al., 2019) and may account for the persistence of drug memories (Sorg et al., 2016). In the cerebellum, PNNs surround Golgi inhibitory interneurons, Lugaro cells, and neurons in the deep cerebellar nuclei (DCN) (Corvetti and Rossi, 2005; Carulli et al., 2006, 2020). Increased cerebellar activity and PNN expression in the cerebellar cortex were exclusive features of animals that expressed CPP and were not observed when cocaine was randomly paired with cues or in saline-treated animals (Carbo-Gas et al., 2017). These results preclude the possibility that cerebellar changes merely result from unconditioned stimulating properties of cocaine or movements performed during the test. In fact, motor activity during the preference test was not different between saline and cocaine-treated groups (Carbo-Gas et al., 2017). Importantly, cerebellar activity and PNN expression correlated with preference for drug-related cues in lobules VIII (LVIII) and IX of the vermis (Carbo-Gas et al., 2014a,^2017^). In addition, the effect of cocaine-induced conditioning on cerebellar activity was not replicated when mice were confined and presented with cocaine-related cues without the possibility of expressing the conditioned response (Carbo-Gas et al., 2017).

Given that granule cell activity has been demonstrated to encode reward, the expectation of reward, reward omission, and conditioned responses (Wagner et al., 2017), our findings might suggest that the granule cell layer makes use of drug-cue associative engrams to tune action selection (Miquel et al., 2020). Distinct granule cells subsets are tuned to combinations of actions and rewards enabling specific subpopulations of Purkinje cells to learn what particular action in a specific context is expected to be rewarded (Wagner et al., 2019; Kostadinov and Häusser, 2022). Nevertheless, it is not clear the extent to which the cerebellar cortex would represent the drug-cue association. Our recent results challenge that the initial encoding of drug-cue associative memory involves the vermis. Decreasing glutamatergic transmission from parallel fibers to Purkinje synapsis using a6Cre-Cacna1a KO mice (Galliano et al., 2013) reduced PNN expression around Golgi interneurons and impaired consolidation but not the acquisition of cocaine-induced CPP (Carbo-Gas et al., 2017). Furthermore, enzymatic digestion of PNNs around Golgi interneurons in the posterior vermis impaired short-term memory of cocaine-induced CPP but did not affect its acquisition (Guarque-Chabrera et al., 2022b). Overall, our results suggest that the drug-induced memory engram could be initially encoded elsewhere and conveyed to the vermis for stabilization.

Previous studies on the role of the cerebellum in Pavlovian conditioning indicate that acquisition and short-term expression of the conditioned motor response rely on specific regions in the cerebellar cortex (Galliano et al., 2013). Electrical (Steinmetz et al., 1989) and optogenetic (Albergaria et al., 2018) stimulation of mossy fibers projecting to the granule cell layer replicates the ability of the conditioned stimulus to trigger conditioned blink responses. In addition, acquisition (Supple and Leaton, 1990), consolidation (Sacchetti et al., 2002), reconsolidation (Sacchetti et al., 2007), and extinction (Utz et al., 2015) of fear conditioning have been shown to involve the vermis. Considering that parallel fibers (granule cell axons) make contacts with dendrites of molecular interneurons (Dizon and Khodakhah, 2011), increased granule cell activity in conditioned animals might enhance the inhibitory effect of molecular interneurons onto Purkinje cells in turn facilitating cerebellar outflow by reducing Purkinje inhibitory control over DCN neurons. Moreover, cocaine may exert an unconditioned effect by reducing Purkinje activity (Jiménez-Rivera et al., 2000).

## Contribution of the cerebellum-infralimbic interactions to drug-induced conditioned preferences

Medial prefrontal cortex dysfunction is a key part of the physiopathology of SUD (McFarland and Kalivas, 2001; Goldstein and Volkow, 2011) and other neuropsychiatric disorders that have shown comorbidity with SUD (Hester and Garavan, 2004; Winstanley et al., 2010; Robbins et al., 2012). More importantly, suffering from schizophrenia, autism, OCD, or ADHD disorder may operate as a risk factor for SUD (Miquel et al., 2019; Ersche et al., 2020). Many of these disorders also involve alterations in the cerebellum (Moulton et al., 2014; Brady et al., 2019; Miquel et al., 2019; Miterko et al., 2019; Kelly et al., 2020). Recently, we intended to approach the study of cerebellar responses when cocaine-induced conditioning is acquired under mPFC impairment (Gil-Miravet et al., 2019; Guarque-Chabrera et al., 2022a). Deactivation of the IL but not PL cortex encourages the acquisition of preference for cocaine-related cues (Guarque-Chabrera et al., 2022a). More importantly, only IL deactivation during drug learning affected neural activity and PNN expression in the posterior cerebellar vermis. IL deactivation increases activity specifically in vGluT2 synapses, but it does not affect vGluT1-related activity. Moreover, it upregulates PNNs particularly around neurogranin + PNN-expressing Golgi cells (Guarque-Chabrera et al., 2022a). These cerebellar hallmarks would result from the interaction between IL impairment and cocaine-induced learning, given that neither of these changes were found when the drug was randomly associated with the contextual cues. A decade ago, we proposed that prefrontal dysfunction would encourage cerebellar hyper-responsiveness if exposure to addictive drugs was repeated (Miquel et al., 2009). Our recent findings support this working hypothesis and suggest that taking drugs when there is a ventral prefrontal dysfunction can cause a functional reorganization of the prefrontal-cerebellar network and boost drug impact on behavior.

Studies over the last decade have established the existence of bidirectional anatomical and functional loops between the mPFC and cerebellum (Watson et al., 2014; Gil-Miravet et al., 2021; Pisano et al., 2021). Cerebellar-mPFC interactions are mediated by the thalamus, basal ganglia, and the VTA (Moers-Hornikx et al., 2009; Watabe-Uchida et al., 2012; Carta et al., 2019). A dopaminergic VTA-cerebellar projection releases detectable dopamine (DA) levels in the posterior lobules of the vermis (VII–X), the right and left hemispheres and the fastigial, interposed, and dentate nuclei (Glaser et al., 2006). In turn, the cerebellar cortex regulates DA activity by several independent pathways. A monosynaptic glutamatergic input from the DCN targets dopaminergic and non-dopaminergic neurons in the VTA (Snider et al., 1976; Watabe-Uchida et al., 2012; Beier et al., 2015; Carta et al., 2019; Gil-Miravet et al., 2021) and projects to the mPFC (Gil-Miravet et al., 2021). In addition, the cerebellum connects to the VTA through the reticulotegmental and pedunculopontine nuclei (Forster and Blaha, 2003) as well as through the mediodorsal, ventrolateral, and ventromedial thalamus (Rogers et al., 2011; Kelly et al., 2020).

At a functional level, it has been evidenced that the cerebellum modulates cerebral cortical functions (Chen et al., 2014; Gao et al., 2018; Kelly et al., 2020; McAfee et al., 2022) and can regulate activity throughout the striatum-cortico-limbic circuitry (Carta et al., 2019; Gil-Miravet et al., 2021; Yoshida et al., 2022). Concurrent activation of the cerebral cortex and the cerebellum potentiates activity and plasticity (LTP/LTD) in the cortico-striatal pathway (Chen et al., 2014). Recently, we have shown that deactivation of the posterior vermis (LVIII) during the acquisition of cocaine-induced CPP dramatically facilitated drug learning and increased neuronal activity in the interposed (Int) and lateral (Lat) DCN, mPFC, NAc, and all striatal subdivisions, except the ventrolateral striatum (Gil-Miravet et al., 2019, 2021). Another effect of deactivating the posterior vermis during drug-induced learning was to enhance PNN expression around GABA/Parvalbumin + interneurons in PL and IL cortices. Interestingly, previous research showed that degradation of PNNs around PL parvalbumin + interneurons prevented cocaine-induced conditioning (Slaker et al., 2015). Therefore, cerebellar cortex disruption in the posterior vermis potentiates neural activity and mechanisms for synaptic stabilization in the addiction circuitry that might facilitate drug-induced learning.

Together, our previous findings indicate that only the IL cortex is functionally related to the cerebellum whereas the cerebellum exerts a modulatory action on the addiction circuitry including both subdivisions of the mPFC (Gil-Miravet et al., 2019, 2021; Guarque-Chabrera et al., 2022a). The cerebellum-IL loop regulates drug effects on behavior in an inhibitory compensatory manner given that impairment of each region is sufficient to enhance neural activity and mechanisms for synaptic stabilization (PNNs) in the other region, potentiating drug-related learning.

**FIGURE 1:**
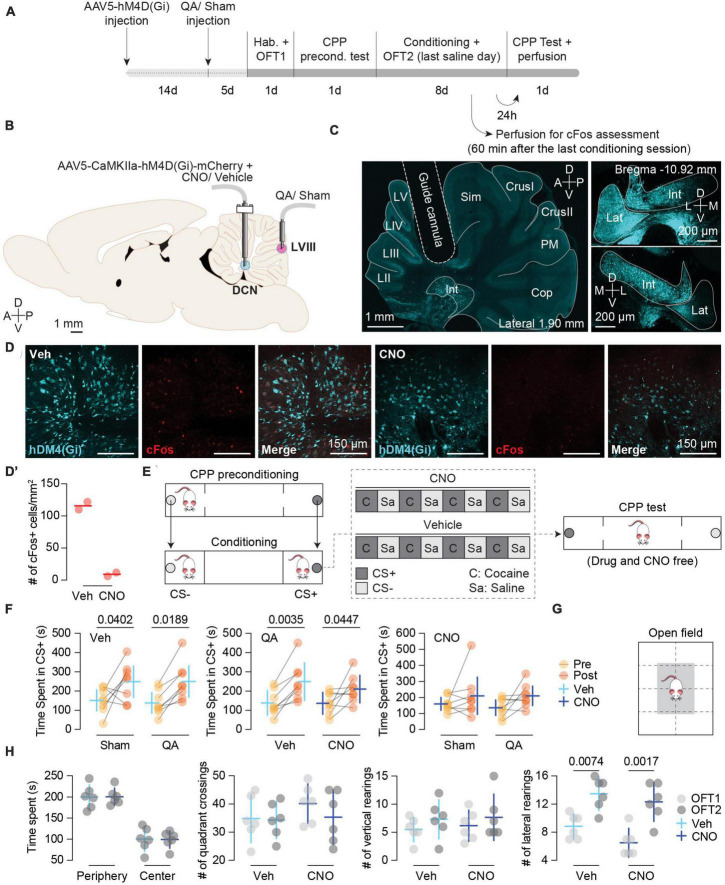
**(A)** Experimental timeline. Different stages of the experimental procedure from stereotaxic surgeries to behavioral protocol. **(B)** Schematic depiction of intracranial infusions and cannula placements. **(C)** DREADD expression and cannula implant. Left panel: tilescan image depicting the guide cannula aiming at a region that encompasses the Interposed (Int) and Lateral (Lat) nuclei where the AAV5-CaMKII-hM4D(Gi) was previously injected showing positive cells expressing the inhibitory DREADD (blue). Right panels: left (top) and right (bottom) DCN images (magnifications extracted from the same section) showing the bilateral expression of AAV5-CaMKII-hM4D(Gi). **(D)** Representative images of cFos expression in the Int nucleus from hM4D(Gi) + Vehicle (Veh) (left) and hM4D(Gi) + CNO (right) rats. Cerebellar tissue was obtained the last conditioning day in which the Veh and CNO animals received a saline injection. CNO reduces neuronal activity in the Int in rats with hM4D(Gi)-mCherry expression (*n* = 2). **(D’)** cFos quantification (*n* = 2). Data are shown as single scores and mean. **(E)** Schematic representation of the CPP apparatus, biased procedure, and conditioning schedule for the Vehicle and CNO groups. **(F)** Pre- and Post-conditioning comparisons of Time spent in the CS + (s) between: Veh-treated animals with Sham (*n* = 8) vs. QA (*n* = 8) lesions (left panel), QA-lesioned animals with Veh (*n* = 8) vs. CNO (*n* = 8) treatments (middle panel), CNO-treated animals with Sham (*n* = 8) vs. QA (*n* = 8) lesion (right panel). Top left label on each plot reports the common treatment of both groups. Left (Veh): Sham *MD*_Pre–Post_ = –96.88 [–189.50, –4.21], *p* = 0.0402; QA *MD*_Pre–Post_ = –111.1 [–203.80, –18.46]; *p* = 0.0189. Middle (QA): Veh *MD*_Pre–Post_ = –111.1 [–183.30, –38.94], *p* = 0.0035; CNO *MD*_Pre–Post_ = –73.88 [–146.10, –1.69], *p* = 0.0447. Data are shown as Mean ± 95% CI. **(G)** Schematic representation of the open field box. **(H)** CNO treatment did not have any effect on motor activity. Left to right: time spent in the periphery [white area in panel **(G)**] and center [gray area in panel **(G)**] of the open field box, number of quadrant crossings, number of vertical rearings, and number of lateral rearings (Lateral rearing: *MD*_OFC1–OFC2_ = –5.25 [–7.20, –3.30], *p* = 0.0001). Vehicle (Veh; *n* = 6); CNO (*n* = 6); OFT1: open field test before conditioning; OFT2: Open field test after 8 days of cocaine-induced conditioning. Data are shown as Mean ± 95% CI.

**FIGURE 2:**
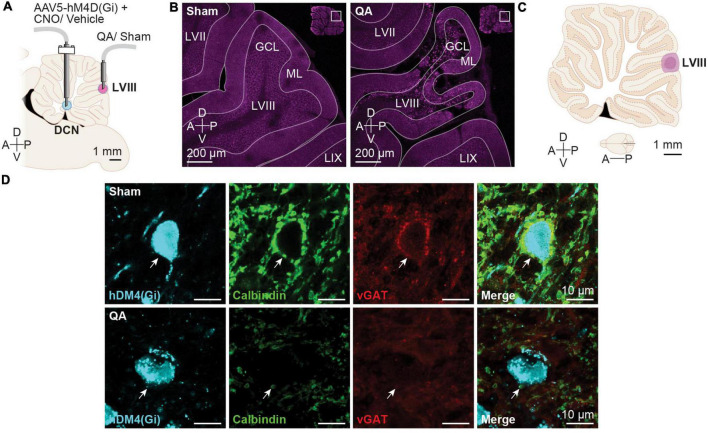
Deep cerebellar nuclei (DCN) effect of sham and quinolinic acid infusions. **(A)** Schematic depiction of intracranial infusions and cannula placements. **(B)** Representative confocal images of Sham and QA injections. **(C)** Schematic diagram depicting the largest (light purple) and smallest (dark purple) diffusion areas in the apical region of LVIII in the vermis. The diffusion areas were established overlaying each cerebellar image over the correspondent rat brain digital atlas image (Paxinos and Watson, 1998). **(D)** Representative images of hM4D(Gi) + cells in the Int nucleus from of Sham- and QA-infused animals. QA reduced the number of Purkinje cell axon terminals (Calbindin +; green) and de vesicular GABA transporter (vGAT +; red) onto DCN neurons expressing the hM4D(Gi)-mCherry DREADD (cyan). White arrows display examples of a Purkinje cell axon terminal.

In the following sections, we present the results of a preliminary study in which we tested the causal link between cerebellar cortex impairment and increased cocaine impact on CPP learning in rats. More specifically, this study used chemogenetic tools to inhibit the activity of a region in the DCN that includes the Int and Lat nuclei (Low et al., 2021). We previously demonstrated that both DCN were targeted by Purkinje terminals from LVIII (Gil-Miravet et al., 2021). We hypothesized that Int and Lat inhibition using DREADDs would prevent the facilitating effect of a posterior vermis lesion on cocaine-induced CPP. We infused quinolinic acid (QA) in LVIII to induce a permanent lesion of this lobule (Gil-Miravet et al., 2019, 2021; Figures 1A, B, 2A–C). To modulate the activity of these DCN, rats were injected bilaterally with the adeno-associated virus expressing the inhibitory DREADD [hM4D(Gi)] and activated the DREADD by a bilateral intracranial injection of Clozapine N-oxide dihydrochloride (CNO) targeting the same regions (Figures 1A–C).

## Preliminary results

### Inhibition of the Int and Lat DCN prevented the facilitating effect of a posterior vermis lesion on cocaine-induced CPP

To test the effectiveness of CNO in inhibiting DCN neural activity, we sampled two AAV5-CaMKIIa-hM4D(Gi)-expressing rats per group and assessed cFos expression. The average number of cFos + cells/mm^2^ was 8.92 [0, 46.72] for CNO-treated (*n* = 2) and 115.90 [40.39, 191.50] for Vehicle (Veh)-treated (*n* = 2) rats (Figures 1A, D, D’).

In two earlier studies, we showed that an excitotoxic lesion with QA in the apical region of LVIII dramatically facilitated the acquisition of cocaine-induced conditioned preference (Gil-Miravet et al., 2019; Gil-Miravet et al., 2021). Then, the facilitation of drug conditioning did not affect the magnitude but the fraction of subjects that expressed the conditioned response. The present results showed a similar effect.

In the present study, we confirmed by immunofluorescence analysis that QA lesion (Figures 2B, C) was effective in reducing Purkinje inputs to DCN neurons (Figure 2D). To verify the facilitation of the acquisition of CPP caused by the vermis lesion, we compared the time spent (TS) in the CS + chamber between the Sham- and QA-lesioned animals with vehicle (Veh) treatment. Both groups increased the TS chamber of the initially non-preferred configuration [Pre/Post test: *F*(1,14) = 15.78, *p* = 0.0014] (Figure 1F, left panel). Moreover, we observed that from all Veh-treated animals, 100% of the QA-lesioned rats increased their TS in the CS + in the post test (Sign test: *PS*_QA_ = 100 [63, 100]%, *p* = 0.0078) whereas only 63% of the Sham-lesioned group displayed higher scores in the second preference test (Sign test: *PS*_Sham_ = 63 [24, 91]%, *p* = 0.7266) (Figure 1F, left panel). Then, we wondered whether CNO treatment in the Int and Lat DCN might influence the effects of a QA lesion in LVIII. To answer this, we compared the Veh and CNO treatments in QA-lesioned animals. This comparison revealed that DCN inhibition did not affect the acquisition of cocaine-induced CPP; all groups acquired a conditioned preference for the initially non-preferred configuration [Pre/Post test: *F*(1,14) = 20.57, *p* = 0.0005] (Figure 1F, middle panel). However, the DCN inhibition using the AAV5-CaMKIIa-hM4D(Gi) seemed to have interfered with the facilitating effect of the QA vermis lesion on drug memory, reducing the proportion of animals that increased their TS in the CS + chamber in the post-conditioning test in the CNO animals (Sign test: *PS*_*CNO*_ = 63 [24, 91]%, *p* = 0.7266) when compared with the Veh group (Sign test: *PS*_Veh_ = 100 [63, 100]%, *p* = 0.0078) (Figure 1F, middle panel).

To confirm that DCN inhibition only prevented the facilitation of the acquisition of CPP caused by the vermis lesion, we compared the TS in the CS + chamber on the post-conditioning test between the Sham and QA groups infused CNO. The QA-CNO group not only spend similar time in the CS + chamber as the Sham-CNO group [Pre/Post test: *F*(1,14) = 4.48, *p* = 0.0532], but also displayed similar probability of change between tests (*PS*_Sham_ = 63 [24, 91]%, *p* = 0.7266; *PS*_QA_ = 63 [24, 91]%, *p* = 0.7266) (Figure 1F; right panel). Moreover, Sham-Veh and -CNO groups showed similar TS in the CS + chamber in the post-conditioning test (*p* = 0.6493; Figure 1F, left and right panels) and proportion of animals increasing their scores in the second test (*PS*_Sham_ = 63 [24, 91]%, *p* = 0.7266). Therefore, Int and Lat inhibition seemed to have only influenced the effect of the vermis lesion (QA) on cocaine-induced CPP (Figure 1F).

### CNO infused in the Int and Lat did not affect motor activity after eight infusions

To rule out a general motor impairment caused by the inhibition of the DCN or intracerebellar infusions of CNO, we tested motor activity in the open field before (OFT1) and after conditioning (OFT2) (*n* = 6) (Figures 1G, H). We did not observe any effect of the treatment in TS in the periphery or center [*F*(1, 10) = 0.00, *p* = 1.000], number of quadrant crossings [*F*(1, 10) = 0.70, *p* = 0.4230], vertical [*F*(1, 10) = 0.11, *p* = 0.7474] or lateral rearing [*F*(1, 10) = 3.74, *p* = 0.0818] (Figure 1H; left to right panels, respectively). However, we did find an effect of the test factor on lateral rearing [*F*(1, 10) = 35.89, *p* = 0.0001] that increased after conditioning (OFT2) regardless of CNO treatment (Figure 1H; right panel).

## Discussion and concluding remarks

Our previous findings showed that a neurotoxic lesion with QA in the apical region of LVIII of the vermis facilitates learning of cocaine-induced conditioned preferences and increases neuronal activity in the Lat nucleus, mPFC, NAc, and all striatal subdivisions, except the ventrolateral striatum (Gil-Miravet et al., 2019, 2021). Using traditional anterograde and retrograde tracing, we also showed that glutamatergic projections from the Interposed and Lateral nuclei to the VTA receive Purkinje axon terminals from LVIII (Gil-Miravet et al., 2021). These findings suggested an inhibitory modulation of the posterior vermis on the activity of the addiction circuitry through the Int and Lateral DCN. This inhibitory control may be compromised under cerebellar vermis dysfunction and potentiates drug effects on behavior (Schmahmann and Sherman, 1998; Miquel et al., 2019).

With the present exploratory study, we aimed to further support this working model using a chemogenetic inhibition of the aDCN. In line with this model, our preliminary findings suggest that DCN inhibition reduces the facilitating effect of a vermis lesion on learning cocaine-induced conditioned preferences. Inhibition of the Int and Lat DCN left intact the ability to acquire conditioned preferences for drug-related stimuli and did not cause any motor impairment or anxiogenic effects. Nevertheless, we did find an increase in lateral rearing after conditioning. This result points to motor sensitization following four cocaine administrations despite having conducted the motor test in a different chamber from that used for conditioning. This effect was not affected either by QA or CNO administrations.

Our QA lesion permanently damages the most external region of the granule cell layer, Purkinje neurons, and molecular layer interneurons in the same region, reducing the inhibitory control of a subpopulation of Purkinje neurons onto DCN neurons. Consequently, lacking Purkinje inhibition, the activity in the cerebellar output neurons and the other regions in the striatum-cortico-limbic circuitry may increase and encourage drug-induced learning (Gil-Miravet et al., 2021). The lesion of LVIII (QA-Veh) increased the proportion of animals that expressed conditioned preference up to 100%, and the inhibition of the DCN decreased the fraction of rats that expressed cocaine-induced conditioning to 63%. Interestingly, our rats still learned to prefer the cocaine-associated configuration despite Int and Lat inhibition, which suggests that the formation of the drug-cue memory trace does not require these cerebellar nuclei. Accordingly, acquisition and expression of eyeblink and fear conditioning do not rely on the DCN but in specific regions of the cerebellar cortex (Supple and Leaton, 1990; Galliano et al., 2013; Carulli et al., 2020). Even so, the DCN may fuel drug memory acquisition when there is posterior vermis dysfunction, hypothetically, through the modulation of activity in the VTA. Transneuronal viral labeling (Watabe-Uchida et al., 2012; Carta et al., 2019) or traditional tracing agent (Gil-Miravet et al., 2021) studies have demonstrated direct glutamatergic projections from the DCN to the VTA that control dopaminergic activity (Carta et al., 2019) and make synaptic contacts with VTA projecting neurons to the mPFC (Gil-Miravet et al., 2021).

Altogether, these results confirm the modulatory role of the cerebellum on cocaine-induced conditioned memory and support the causal link between vermis dysfunction and the facilitation of drug effects.

It has been hypothesized that the cerebellar modulation consists of what has been called “cerebellar brain inhibitory function” (Darch et al., 2018). Patients with posterior cerebellum impairment, particularly in the vermis, show difficulties in controlling their behavior, emotions, and cognitions in what has been called “the cerebellar cognitive-affective syndrome” (Schmahmann and Sherman, 1998). Dysregulation of the cerebellar vermis has been also proposed as a potential etiological factor for ADHD and other disorders of the impulsive spectrum (Mulder et al., 2008; Durston et al., 2011; Miquel et al., 2019) that result in comorbidity to SUD. Therefore, the present findings may open new avenues to consider the cerebellum as a therapeutic target for stimulation in SUD and other comorbid mental disorders such as ADHD.

## Methods

### Subjects

The present study includes 36 (*n* = 32 in experiment 1; *n* = 4 in experiment 2) male Sprague-Dawley rats weighing 125–225 g (Janvier, ST Berthevin Cedex, France). Rats were housed individually in the animal facilities (Jaume I University, Spain) under standard conditions (12-h light cycle from 8:00 a.m. to 8:00 p.m., with access to food and water ad *libitum*). Before beginning with behavioral procedures, rats were handled for 3 weeks and habituated to experimental protocols. Animal procedures were approved by the local Animal Welfare Ethics Committee and Empowered Body (2014/VSC/PEA/00208; 0139) and adhered to the European Community Council directive (2010/63/EU), Spanish directive BOE 34/11370/2013, and local directive DOGV 26/2010.

### Pharmacological agents, adenoviruses, and DREADDs

Cocaine hydrochloride (Alcaliber S.A., Madrid, Spain) was dissolved in a 0.90% saline solution and administered intraperitoneally (IP). Saline solution was used as a control vehicle. Anesthesia was induced with isoflurane (1,000 mg/g) (Isoflutek 250 ml, Laboratorios Karizoo S.A., Barcelona, Spain) (induction at 3.00% and maintenance through the whole surgery at 2.00%) using a Isotec 5 isoflurane anesthesia vaporizer (Datex-Ohmeda Inc., Madison, WI, USA). AAV5-CaMKIIa-hM4D(Gi)-mCherry (Titer ≥ 3 × 10^12^ vg/mL; Cat# 50477) used in the present study were provided by Addgene (Watertown, MA, USA). DREADD activation was achieved using 1 mM intracranial 0.50 μL/side and 1 mg/kg intraperitoneal of Clozapine N-oxide dihydrochloride (CNO-100 mg, Hello Bio Inc., Princeton, USA; Cat# HB6149).

### Intracranial injections

Rats weighing between 270 and 350 g were anesthetized with isoflurane (1,000 mg/g) (induction at 3.00% and maintenance through the whole surgery at 2.00%) using an Isotec 5 isoflurane anesthesia vaporizer and placed in a stereotaxic apparatus (Kopf Model 902; David Kopf Instruments, Tujunga, CA, USA).

In the first stereotaxic surgery, intracranial infusions of AAV were performed by placing a stainless steel guide cannula (23-gauge external diameter, 36 mm length) in the Int (AP: −11.30; ML: ± 2.50; DV: −5.80) (Paxinos and Watson, 1998; Figures 1A, B). Then, a removable stainless-steel injector (30-gauge external diameter, 38 mm length) connected to a 1 μL Hamilton syringe (Microliter Syringe Model 7102 KH; Cat# 80300) driven by an infusion pump (11 Plus dual Syringe; Harvard Apparatus; Cat# HA702209) was inserted into the previously placed guide cannula to infuse bilaterally 0.50 μL of AVV (0.50 μL/min, two infusions of 0.25 μL separated by a period of 3 min) for the viral expression. After the infusion completion, the injector remained in place for 3 min for a better absorption of the substance. Then, the guide cannula and injector were removed, and the wound was sutured (Figures 1A, B). DREADD expression was observed in the Int and Lat DCN.

After 2 weeks from the AVV infusion, a second stereotactic surgery was performed to infuse in the posterior cerebellum (LVIII) (AP: −14.50; ML: 0.00; DV: −4.50) 0.50 μL (0.50 μL/min) of QA (90 nmol/μL) (2,3-pyridinedicarboxylic acid, Sigma-Aldrich, Madrid, Spain; Cat# P63204) dissolved in phosphate-buffered saline (PBS; 0.10M pH 7.40) in order to permanently inactivate the apical region of vermis LVIII (Gil-Miravet et al., 2019, 2021; Figures 1A, B, 2A–C). After the infusion was completed, the injector remained in place for 5 min to avoid liquid aspiration. Then, the guide cannula and injector were removed. In the same surgery, for future infusion of the DREADD activator (CNO) or vehicle (saline), two guide cannulas (30-gauge external diameter, 15.20 mm length) were aimed bilaterally at the Int and attached to the skull with two stainless steel screws and fixed with dental cement (Figures 1A, B). Then, a dummy cannula (23-gauge external diameter, 15.20 mm length) was placed and kept inside the guide cannula to maintain the cannula’s integrity until experiments were performed. After surgery, animals received analgesic treatment with meloxicam (Metacam 20 mL, 5 mg/mL; Boehringer Ingelheim, Barcelona, Spain, CAT#9993012) every 24 h for 2 days. Rats were left undisturbed for 4 days for recovery. For behavioral experiments with intracranial infusions, rats were gently handled while restrained, and CNO or saline were infused bilaterally into the Int 5 min before each training session (0.50 μL, at 0.50 μL/min). Rats were not anesthetized during the microinjections because this procedure does not involve pain or discomfort for the animals. Behavioral trials began 5 min after the infusion.

### Conditioned place preference

This procedure was conducted in an opaque, oblong corridor (90 × 20 × 60 cm) that included two lateral black chambers (20 × 20 × 60 cm) located on opposite sides. Two days before the training, animals were habituated to the apparatus in a 15-min session without texturized floor (stainless-steel floor with perforated small holes or big holes) (Leroy Merlin SL., Alcobendas, Spain) or olfactory cues (lemon or almond) (Gran Velada SL., Zaragoza, Spain). For the preconditioning preference test (Figure 1E), rats were placed in the central chamber and were free to move throughout the corridor in a 20 min drug-free test in which the two olfactory and tactile cues configurations (lemon + big holes or almond + small holes) were presented simultaneously but in opposite arms in the corridor. All the test sessions were videotaped and scored by a blind observer. The first 5 min were not considered to allow the animal to explore the location of the odors. In the conditioning phase (Figure 1E), the least preferred stimulus configuration for each animal acted as CS + and was paired with IP cocaine (10 mg/kg). On alternate days, the other configuration was associated with IP saline injections (CS-). For each pairing session, rats were confined in one of the lateral chambers of the apparatus for 25 min. Therefore, four cocaine- and four saline-paired sessions were conducted on alternate days (Figure 1E). The preference test was identical to the preconditioning test and was carried out 24 h after the last conditioning session and 48 h after the last cocaine administration (Figure 1E). The location of the cues in the corridor was counterbalanced between animals.

### Open field test

We performed this test during habituation (before training) and the last saline-paired day (after training but without CNO; without Int inactivation). Rats were placed in the center of a 60 × 40 × 30 cm acrylic box for 15 min (Figure 1G). The open field was cleaned with 20% ethanol before each test to avoid any possible odor contamination that might affect the experimental result. All tests were videotaped, and spontaneous locomotor activity was evaluated blindly, assessing time spent in the periphery/center, number of quadrant crossings, and vertical and lateral rearing (Figure 1H). These parameters were recorded in three blocks of 5 min. Only the intermedium 5 min block is represented in figures to avoid confounding effects such as initial exploration and boredom at the end of the period.

### Brain sampling

All animals were perfused transcardially 60 min following the last preference test on each behavioral protocol using saline (0.90%), heparine (0.006%) (Heparin sodium salt from porcine intestinal mucosa; CAT# H3393; Sigma-Aldrich, Madrid, Spain) and then paraformaldehyde (4%) (Paraformaldehyde powder, 95%; CAT#158127; Sigma-Aldrich, Madrid, Spain). And paraformaldehyde (4%) under anesthesia with sodium pentobarbital (30 mg/kg) (Dolethal 100 mL, Vetoquinol E.V.S.A., Madrid, Spain). Brains were extracted and stored with the same fixative for 24 h at 4°C. Then, the tissue was immersed in sucrose solution (30%) with sodium azide (2%) (Sodium azide; Cat# 1.06688.0100; Merck, Darmstadt, Germany) until the brain sank at 4°C. Depending on the region of interest, sagittal or coronal sections were obtained at 40 μm with a cryostat microtome (Microm HM560, Thermo Fisher Scientific, Barcelona, Spain) and stored at −22°C in cryoprotectant solution. The expression of the AAV5-CaMKIIa-hM4D(Gi) was checked using confocal imaging. Animals with no DREADD expression or cannula misplacement were not included in the statistical analysis.

### Immunofluorescence and imaging

Immunolabeling was performed on free-floating sections. To amplify the endogenous mCherry fluorescent signal, sections were exposed to a blocking buffer with donkey serum (Sigma Aldrich, Madrid, Spain; Cat# D9663) for 30 min and then incubated overnight at 4°C in PBS 0.1 M Triton X-100 (Sigma-Aldrich, Madrid, Spain; Cat# T9284) (PBST) with rabbit anti-RFP (1:500; Rockland Immunochemicals Inc., Philadelphia, PA, USA; Cat# 600-401-379). The next day, and after several rinses, tissue was incubated in PBST with anti-rabbit Alexa Fluor 555 (1:500; Thermo Fisher Scientific, Rockford, IL, USA; Cat# A-31572) as secondary antibody. We used cFos immunofluorescent staining as a neural activity marker. Similarly, sections were incubated for 48 h at 4°C with rabbit anti-cFos (1:500; Synaptic Systems, Goettingen, Germany; Cat# 226003) in PBST, and the next day, in PBST with donkey anti-rabbit Alexa Fluor 647 (1:500; Thermo Fisher Scientific, Rockford, IL, USA; Cat# A-31573) as secondary antibody. To ensure that the QA lesion was affecting Purkinje innervation in the DCN, we used Calbindin as a marker for Purkinje terminals and vGAT to estimate GABA-related activity. Sections were incubated for 24 h at 4°C with guinea pig anti-vGAT (1:500; Synaptic Systems, Goettingen, Germany; Cat# 131004) and mouse anti-CB (1:1000 Swant, Bellinzona, Switzerland; Cat# CB38) in PBST, and the next day, in PBST with donkey anti-guinea pig Alexa Fluor 647 (1:250; Thermo Fisher Scientific, Rockford, IL, USA; Cat# A-21450) and donkey anti-mouse Cy3 (1:200 Jackson ImmunoResearch, West Grove, PA, USA; Cat# 715-165-150) as secondary antibodies. Brain tissue was mounted and covered with Mowiol (Calbiochem, Merck Chemicals and Life Science, Madrid, Spain; Cat# 475904100GM).

### Image acquisition and analysis

Laser intensity, gain, and offset remained stable across animals. Tile-scan Z-stack images and image magnifications of the posterior cerebellum or DCN were acquired using confocal microscopy (Leica DMi8, Leica Microsystems CMS GmbH, Wetzlar, Germany) to assess QA/Sham lesions, cannula placements, and DREADD/mCherry expression. Imaging for cFos was perfumed at 20×, and vGAT and Calbindin co-expression around DREADD-expressing DCN neurons was performed at 63×. We used Leica Application Suite X (LAS X, Leica Microsystems CMS GmbH, Wetzlar, Germany) to perform maximal projections of the Z-stacks and Fiji free software (Schindelin et al., 2012) for image analysis. cFos + cells (intense red labeling) were estimated in 2 images per rat (*n* = 2) co-expressing the AAV5-CaMKIIa-hM4D(Gi) within a ROI of 580 × 580 μm.

### Experimental design and statistics

We used a total sample size of 32 animals, 8 animals per group (Veh-Sham, Veh-QA, CNO-Sham, CNO-QA). Statistical analyses were performed using GraphPad Prism 9 software (GraphPad Software Inc., La Jolla, CA, USA), and R Statistical language (version 4.1.2; R Core Team, 2021). Data were analyzed by repeated-measures two-way ANOVAs and multiple comparisons were performed using Sidak’s tests. When two groups were compared, we used an unpaired *t*-test. Data are presented as individual and group scores (Mean and 95% Confidence Intervals; 95% CI). In the cases in which a significant difference was observed, we calculated the effect size of that difference as the Mean difference (*MD* = Mean Group1 – Mean Group2) and its 95% CI, to estimate the magnitude of those differences.

To assess for consistent differences between the weight of subjects before and after conditioning, we used the sign tests (Two-sided sign test for matched pairs) to assess the proportion of animals whose TS in the CS + increases (probability of success) with respect to themselves in the previous test. Then we assessed whether that probability of change as a group was different or not from chance (0.5) using the *binom.test* of the R *mosaic* package (Pruim et al., 2017), that returns the probability of success (as a group) with its 95% Confidence Interval (CI) and exact *p*-value (having as an alternative hypothesis: true probability of success is not equal to 0.5), which is what was reported.

## Data availability statement

The raw data supporting the conclusions of this article will be made available by the authors, without undue reservation.

## Ethics statement

This animal study was reviewed and approved by the Local Animal Welfare Ethics Committee and Empowered Body (2014/VSC/PEA/00208; 0139; 2019/VSC/PEA0252).

## Author contributions

MM and RP: conceptualization. IM-E and MM: methodology. IM-E, JG-C, MM, AS-H, and PI-M: formal and statistical analysis. IM-E: investigation. IM-E, MM, and RP: writing—original draft. IM-E, JG-C, MM, RP, AS-H, and PI-M: writing—review and editing. JG-C, IM-E, AS-H, and PI-M: visualization. MM: funding acquisition. All authors full access to all data, take responsibility for all the information in the present manuscript, made a notable contribution to the manuscript, were involved in critically revising the present version, and approved the present version of the manuscript.

## References

[B1] Albergaria C. Silva N. T. Pritchett D. L. Carey M. R. (2018). Locomotor activity modulates associative learning in mouse cerebellum. *Nat. Neurosci.* 21 725–735. 10.1038/s41593-018-0129-x 29662214 PMC5923878

[B2] Al-Khalil K. Vakamudi K. Witkiewitz K. Claus E. D. (2021). Neural correlates of alcohol use disorder severity among nontreatment-seeking heavy drinkers: An examination of the incentive salience and negative emotionality domains of the alcohol and addiction research domain criteria. *Alcohol. Clin. Exp. Res.* 45 1200–1214. 10.1111/acer.14614 33864389

[B3] Anderson C. M. Maas L. C. Frederick B. Bendor J. T. Spencer T. J. Livni E. (2006). Cerebellar vermis involvement in cocaine-related behaviors. *Neuropsychopharmacology* 31 1318–1326. 16237382 10.1038/sj.npp.1300937

[B4] Arnsten A. F. T. (2006). Fundamentals of attention- deficit/hyperactivity disorder: Circuits and pathways. *J. Clin. Psychiatry* 67 7–12. 16961424

[B5] Bardo M. T. Bevins R. A. (2000). Conditioned place preference: What does it add to our preclinical understanding of drug reward? *Psychopharmacology (Berl.)* 153 31–43. 10.1007/s002130000569 11255927

[B6] Becker E. B. E. Stoodley C. J. (2013). “Autism spectrum disorder and the cerebellum,” in *International review of neurobiology*, 1st Edn, ed. Konopka G. (Amsterdam: Elsevier Inc). 10.1016/B978-0-12-418700-9.00001-0 24290381

[B7] Beier K. T. Steinberg E. E. DeLoach K. E. Xie S. Miyamichi K. Schwarz L. (2015). Circuit architecture of VTA dopamine neurons revealed by systematic input-output mapping. *Cell* 162 622–634. 10.1016/j.cell.2015.07.015 26232228 PMC4522312

[B8] Bonson K. R. Grant S. J. Contoreggi C. S. Links J. M. Metcalfe J. Weyl H. L. (2002). Neural systems and cue-induced cocaine craving. *Neuropsychopharmacology* 26 376–386. 11850152 10.1016/S0893-133X(01)00371-2

[B9] Bostan A. C. Strick P. L. (2018). The basal ganglia and the cerebellum: Nodes in an integrated network. *Nat. Rev. Neurosci.* 19 338–350. 10.1038/s41583-018-0002-7 29643480 PMC6503669

[B10] Bostan A. C. Dum R. P. Strick P. L. (2010). The basal ganglia communicate with the cerebellum. *Proc. Natl. Acad. Sci. U.S.A.* 107 8452–8456. 10.1073/pnas.1000496107 20404184 PMC2889518

[B11] Brady R. O. Jr. Gonsalvez I. Lee I. Öngür D. Seidman L. J. Schmahmann J. D. (2019). Cerebellar-prefrontal network connectivity and negative symptoms in schizophrenia. *Am. J. Psychiatry* 176 512–520. 10.1176/appi.ajp.2018.18040429 30696271 PMC6760327

[B12] Carbo-Gas M. Moreno-Rius J. Guarque-Chabrera J. Vazquez-Sanroman D. Gil-Miravet I. Carulli D. (2017). Cerebellar perineuronal nets in cocaine-induced pavlovian memory: Site matters. *Neuropharmacology* 125 166–180. 10.1016/j.neuropharm.2017.07.009 28712684

[B13] Carbo-Gas M. Vazquez-Sanroman D. Aguirre-Manzo L. Coria-Avila G. A. Manzo J. Sanchis-Segura C. (2014a). Involving the cerebellum in cocaine-induced memory: Pattern of CFos expression in mice trained to acquire conditioned preference for cocaine. *Addict. Biol.* 19 61–76. 10.1111/adb.12042 23445190

[B14] Carbo-Gas M. Vazquez-Sanroman D. Gil-Miravet I. De las Heras-Chanes J. Coria-Avila G. A. Manzo J. (2014b). Cerebellar hallmarks of conditioned preference for cocaine. *Physiol. Behav.* 132 24–35. 10.1016/j.physbeh.2014.04.044 24813699

[B15] Carta I. Chen C. H. Schott A. L. Dorizan S. Khodakhah K. (2019). Cerebellar modulation of the reward circuitry and social behavior. *Science* 363:eaav0581. 10.1126/science.aav0581 30655412 PMC6711161

[B16] Carulli D. Broersen R. de Winter F. Muir E. M. Mešković M. de Waal M. (2020). Cerebellar plasticity and associative memories are controlled by perineuronal nets. *Proc. Natl. Acad. Sci. U.S.A.* 117 6855–6865. 10.1073/pnas.1916163117 32152108 PMC7104182

[B17] Carulli D. Rhodes K. E. Brown D. J. Bonnert T. P. Pollack S. J. Oliver K. (2006). Composition of perineuronal nets in the adult rat cerebellum and the cellular origin of their components. *J. Comp. Neurol.* 494 559–577. 10.1002/cne.20822 16374793

[B18] Chen C. H. Fremont R. Arteaga-Bracho E. E. Khodakhah K. (2014). Short latency cerebellar modulation of the basal ganglia. *Nat. Neurosci.* 17 1767–1775. 10.1038/nn.3868 25402853 PMC4241171

[B19] Corvetti L. Rossi F. (2005). Degradation of chondroitin sulfate proteoglycans induces sprouting of intact purkinje axons in the cerebellum of the adult rat. *J. Neurosci.* 25 7150–7158. 10.1523/JNEUROSCI.0683-05.2005 16079397 PMC6725229

[B20] Cunningham C. L. Gremel C. M. Groblewski P. A. (2006). Drug-induced conditioned place preference and aversion in mice. *Nat. Protoc.* 1 1662–1670. 10.1038/nprot.2006.279 17487149

[B21] D’Ambra A. F. Jung S. J. Ganesan S. Antzoulatos E. G. Fioravante D. (2023). Cerebellar activation bidirectionally regulates nucleus accumbens medial shell and core. *bioRxiv* [Preprint]. 10.1101/2020.09.28.283952

[B22] Darch H. T. Cerminara N. L. Gilchrist I. D. Apps R. (2018). “Non-invasive stimulation of the cerebellum in health and disease,” in *Transcranial magnetic stimulation in neuropsychiatry*, Vol. 12 ed. Ustohal L. (London: Intech). 10.5772/intechopen.73218

[B23] De Pirro S. Galati G. Pizzamiglio L. Badiani A. (2018). The affective and neural correlates of heroin versus cocaine use in addiction are influenced by environmental setting but in opposite directions. *J. Neurosci.* 38 5182–5195. 10.1523/JNEUROSCI.0019-18.2018 29760180 PMC6705939

[B24] Dizon M. J. Khodakhah K. (2011). The role of interneurons in shaping Purkinje cell responses in the cerebellar cortex. *J. Neurosci.* 31 10463–10473. 21775592 10.1523/JNEUROSCI.1350-11.2011 PMC3314287

[B25] Durston S. van Belle J. de Zeeuw P. (2011). Differentiating frontostriatal and fronto-cerebellar circuits in attention-deficit/hyperactivity disorder. *Biol. Psychiatry* 69 1178–1184. 10.1016/j.biopsych.2010.07.037 20965496

[B26] Ersche K. D. Meng C. Ziauddeen H. Stochl J. Williams G. B. Bullmore E. T. (2020). Brain networks underlying vulnerability and resilience to drug addiction. *Proc. Natl. Acad. Sci. U.S.A.* 117 15253–15261. 10.1073/pnas.2002509117 32541059 PMC7334452

[B27] Everitt B. J. Robbins T. W. (2005). Neural systems of reinforcement for drug addiction: From actions to habits to compulsion. *Nat. Neurosci.* 8 1481–1489. 10.1038/nn1579 16251991

[B28] Fawcett J. W. Oohashi T. Pizzorusso T. (2019). The roles of perineuronal nets and the perinodal extracellular matrix in neuronal function. *Nat. Rev. Neurosci.* 20 451–465. 10.1038/s41583-019-0196-3 31263252

[B29] Filbey F. M. Schacht J. P. Myers U. S. Chavez R. S. Hutchison K. E. (2009). Marijuana craving in the brain. *Proc. Natl. Acad. Sci. U.S.A.* 106 13016–13021. 19651613 10.1073/pnas.0903863106 PMC2716383

[B30] Forster G. L. Blaha C. D. (2003). Pedunculopontine tegmental stimulation evokes striatal dopamine efflux by activation of acetylcholine and glutamate receptors in the midbrain and pons of the rat. *Eur. J. Neurosci.* 17 751–762. 10.1046/j.1460-9568.2003.02511.x 12603265

[B31] Frontera J. L. Baba Aissa H. Sala R. W. Mailhes-Hamon C. Georgescu I. A. Léna C. (2020). Bidirectional control of fear memories by cerebellar neurons projecting to the ventrolateral periaqueductal grey. *Nat. Commun.* 11:5207. 10.1038/s41467-020-18953-0 33060630 PMC7566591

[B32] Galliano E. De Zeeuw C. I. (2014). Questioning the cerebellar doctrine. *Prog. Brain Res.* 210 59–77. 10.1016/B978-0-444-63356-9.00003-0 24916289

[B33] Galliano E. Gao Z. Schonewille M. Todorov B. Simons E. Pop A. S. (2013). Silencing the majority of cerebellar granule cells uncovers their essential role in motor learning and consolidation. *Cell Rep.* 3 1239–1251. 10.1016/j.celrep.2013.03.023 23583179

[B34] Gao Z. Davis C. Thomas A. M. Economo M. N. Abrego A. M. Svoboda K. (2018). A cortico-cerebellar loop for motor planning. *Nature* 563 113–116. 10.1038/s41586-018-0633-x 30333626 PMC6212318

[B35] Gil-Miravet I. Guarque-Chabrera J. Carbo-Gas M. Olucha-Bordonau F. Miquel M. (2019). The role of the cerebellum in drug-cue associative memory: Functional interactions with the medial prefrontal cortex. *Eur. J. Neurosci.* 50 2613–2622. 10.1111/ejn.14187 30280439

[B36] Gil-Miravet I. Melchor-Eixea I. Arias-Sandoval E. Vasquez-Celaya L. Guarque-Chabrera J. Olucha-Bordonau F. (2021). From back to front: A functional model for the cerebellar modulation in the establishment of conditioned preferences for cocaine-related cues. *Addict. Biol.* 26 e12834. 10.1111/adb.12834 31808992

[B37] Glaser P. E. Surgener S. P. Grondin R. Gash C. R. Palmer M. Castellanos F. X. (2006). Cerebellar neurotransmission in attention-deficit/hyperactivity disorder: Does dopamine neurotransmission occur in the cerebellar vermis? *J. Neurosci. Methods* 151 62–67. 10.1016/j.jneumeth.2005.09.019 16451810

[B38] Goldstein R. Z. Volkow N. D. (2011). Dysfunction of the prefrontal cortex in addiction: Neuroimaging findings and clinical implications. *Nat. Rev. Neurosci.* 12 652–669. 10.1038/nrn3119 22011681 PMC3462342

[B39] Grant S. London E. D. Newlin D. B. Villemagne V. L. Liu X. Contoreggi C. (1996). Activation of memory circuits during cue-elicited cocaine craving. *Proc. Natl. Acad. Sci. U.S.A.* 93 12040–12045. 8876259 10.1073/pnas.93.21.12040 PMC38179

[B40] Grimpe B. Silver J. (2002). The extracellular matrix in axon regeneration. *Prog. Brain Res.* 137 333–349. 10.1016/s0079-6123(02)37025-0 12440376

[B41] Guarque-Chabrera J. Sanchez-Hernandez A. Ibáñez-Marín P. Melchor-Eixea I. Miquel M. (2022b). Role of Perineuronal nets in the cerebellar cortex in cocaine-induced conditioned preference, extinction, and reinstatement. *Neuropharmacology* 218:109210. 10.1016/j.neuropharm.2022.109210 35985392

[B42] Guarque-Chabrera J. Gil-Miravet I. Olucha-Bordonau F. Melchor-Eixea I. Miquel M. (2022a). When the front fails, the rear wins. Cerebellar correlates of prefrontal dysfunction in cocaine-induced memory in male rats. *Prog. Neuropsychopharmacol. Biol. Psychiatry* 112:110429. 10.1016/j.pnpbp.2021.110429 34416354

[B43] Hartmann T. Vandborg S. Rosenberg R. Sørensen L. Videbech P. (2016). Increased fractional anisotropy in cerebellum in obsessive- compulsive disorder. *Acta Neuropsychiatr.* 28 141–148. 10.1017/neu.2015.57 26522275

[B44] Hazari N. Narayanaswamy J. C. Venkatasubramanian G. (2019). Neuroimaging findings in obsessive–compulsive disorder: A narrative review to elucidate neurobiological underpinnings. *Indian J. Psychiatry* 61 S9–S29. 10.4103/psychiatry.IndianJPsychiatry_525_18 30745673 PMC6343409

[B45] Heffley W. Hull C. (2019). Classical conditioning drives learned reward prediction signals in climbing fibers across the lateral cerebellum. *Elife* 8:e46764. 10.7554/eLife.46764 31509108 PMC6845228

[B46] Hester R. Garavan H. (2004). Executive dysfunction in cocaine addiction: Evidence for discordant frontal, cingulate, and cerebellar activity. *J. Neurosci.* 24 11017–11022. 10.1523/JNEUROSCI.3321-04.2004 15590917 PMC6730277

[B47] Holloway Z. R. Paige N. B. Comstock J. F. Nolen H. G. Sable H. J. Lester D. B. (2019). Cerebellar modulation of mesolimbic dopamine transmission is functionally asymmetrical. *Cerebellum* 18 922–931. 10.1007/s12311-019-01074-w 31478166

[B48] Hyman S. E. Malenka R. C. Nestler E. J. (2006). Neural mechanisms of addiction: The role of reward-related learning and memory. *Annu. Rev. Neurosci.* 29 565–598. 10.1146/annurev.neuro.29.051605.113009 16776597

[B49] Jiménez-Rivera C. A. Segarra O. Jiménez Z. Waterhouse B. D. (2000). Effects of intravenous cocaine administration on cerebellar Purkinje cell activity. *Eur. J. Pharmacol.* 407 91–100. 10.1016/s0014-2999(00)00711-1 11050295

[B50] Kelly E. Meng F. Fujita H. Morgado F. Kazemi Y. Rice L. C. (2020). Regulation of autism-relevant behaviors by cerebellar-prefrontal cortical circuits. *Nat. Neurosci.* 23 1102–1110. 10.1038/s41593-020-0665-z 32661395 PMC7483861

[B51] Kilts C. D. Egan G. Gideon D. A. Ely T. D. Hoffman J. M. (2003). Dissociable neural pathways are involved in the recognition of emotion in static and dynamic facial expressions. *Neuroimage* 18 156–168. 10.1006/nimg.2002.1323 12507452

[B52] Kostadinov D. Häusser M. (2022). Reward signals in the cerebellum: Origins, targets, and functional implications. *Neuron* 110 1290–1303. 10.1016/j.neuron.2022.02.015 35325616

[B53] Lou M. Wang E. Shen Y. Wang J. (2012). Cue-elicited craving in heroin addicts at different abstinent time: An fMRI pilot study. *Subst. Use Misuse* 47 631–639. 10.3109/10826084.2011.646381 22329835 PMC3359800

[B54] Low A. Y. T. Goldstein N. Gaunt J. R. Huang K. P. Zainolabidin N. Yip A. K. K. (2021). Reverse-translational identification of a cerebellar satiation network. *Nature* 600 269–273. 10.1038/s41586-021-04143-5 34789878 PMC8665128

[B55] Martin-Sölch C. Magyar S. Künig G. Missimer J. Schultz W. Leenders K. (2001). Changes in brain activation associated with reward processing in smokers and nonsmokers. *Exp. Brain Res.* 139 278–286. 11545466 10.1007/s002210100751

[B56] McAfee S. S. Liu Y. Sillitoe R. V. Heck D. H. (2022). Cerebellar coordination of neuronal communication in cerebral cortex. *Front. Syst. Neurosci.* 15:781527. 10.3389/fnsys.2021.781527 35087384 PMC8787113

[B57] McFarland K. Kalivas P. W. (2001). The circuitry mediating cocaine-induced reinstatement of drug-seeking behavior. *J. Neurosci.* 21 8655–8663. 10.1523/JNEUROSCI.21-21-08655.2001 11606653 PMC6762812

[B58] McKendrick G. Graziane N. M. (2020). Drug-induced conditioned place preference and its practical use in substance use disorder research. *Front. Behav. Neurosci.* 14:582147. 10.3389/fnbeh.2020.582147 33132862 PMC7550834

[B59] Miquel M. Gil-Miravet I. Guarque-Chabrera J. (2020). The cerebellum on cocaine. *Front. Syst. Neurosci.* 14:586574. 10.3389/fnsys.2020.586574 33192350 PMC7641605

[B60] Miquel M. Nicola S. M. Gil-Miravet I. Guarque-Chabrera J. Sanchez-Hernandez A. (2019). A working hypothesis for the role of the cerebellum in impulsivity and compulsivity. *Front. Behav. Neurosci.* 13:99. 10.3389/fnbeh.2019.00099 31133834 PMC6513968

[B61] Miquel M. Toledo R. García L. I. Coria-Avila G. A. Manzo J. (2009). Why should we keep the cerebellum in mind when thinking about addiction? *Curr. Drug Abuse Rev.* 2 26–40. 10.2174/1874473710902010026 19630735

[B62] Miquel M. Vazquez-Sanroman D. Carbo-Gas M. Gil-Miravet I. Sanchis-Segura C. Carulli D. (2016). Have we been ignoring the elephant in the room? Seven arguments for considering the cerebellum as part of addiction circuitry. *Neurosci. Biobehav. Rev.* 60 1–11. 10.1016/j.neubiorev.2015.11.005 26602022

[B63] Miterko L. N. Baker K. B. Beckinghausen J. Bradnam L. V. Cheng M. Y. Cooperrider J. (2019). Consensus paper: Experimental neurostimulation of the cerebellum. *Cerebellum* 6 1064–1097. 10.1007/s12311-019-01041-5 31165428 PMC6867990

[B64] Moers-Hornikx V. M. Sesia T. Basar K. Lim L. W. Hoogland G. Steinbusch H. W. (2009). Cerebellar nuclei are involved in impulsive behaviour. *Behav. Brain Res.* 203 256–263. 10.1016/j.bbr.2009.05.011 19450624

[B65] Moreno-Rius J. Miquel M. (2017). The cerebellum in drug craving. *Drug Alcohol Depend.* 173 151–158. 10.1016/j.drugalcdep.2016.12.028 28259088

[B66] Moulton E. A. Elman I. Becerra L. R. Goldstein R. Z. Borsook D. (2014). The cerebellum and addiction: Insights gained from neuroimaging research. *Addict. Biol.* 19 317–331. 10.1111/adb.12101 24851284 PMC4031616

[B67] Mulder M. J. Baeyens D. Davidson M. C. Casey B. J. Den Ban E. V. Van Engeland H. (2008). Familial vulnerability to ADHD affects activity in the cerebellum in addition to the prefrontal systems. *J. Am. Acad. Child Adolesc. Psychiatry* 47 68–75. 10.1097/chi.0b013e31815a56dc 18174827

[B68] Olbrich H. M. Valerius G. Paris C. Hagenbuch F. Ebert D. Juengling F. D. (2006). Brain activation during craving for alcohol measured by positron emission tomography. *N. Z. J. Psychiatry* 40 171–178. 10.1080/j.1440-1614.2006.01765.x 16476136

[B69] Paxinos G. Watson C. (1998). *The rat brain in stereotaxic coordinates.* San Diego, CA: Academic Press.

[B70] Pisano T. J. Dhanerawala Z. M. Kislin M. Bakshinskaya D. Engel E. A. Hansen E. J. (2021). Homologous organization of cerebellar pathways to sensory, motor, and associative forebrain. *Cell Rep.* 36:109721. 10.1016/j.celrep.2021.109721 34551311 PMC8506234

[B71] Pruim R. Kaplan D. T. Horton N. J. (2017). The mosaic package: Helping students to “think with data” using R. *R J.* 9 77–102. 10.32614/rj-2017-024

[B72] Qing X. Gu L. Li D. (2021). Abnormalities of localized connectivity in obsessive-compulsive disorder: A voxel-wise meta-analysis. *Front. Hum. Neurosci.* 15:739175. 10.3389/fnhum.2021.739175 34602998 PMC8481585

[B73] R Core Team (2021). *R: A language and environment for statistical computing.* Vienna: R Foundation for Statistical Computing.

[B74] Risinger R. C. Salmeron B. J. Ross T. J. Amen S. L. Sanfilipo M. Hoffmann R. G. (2005). Neural correlates of high and craving during cocaine self-administration using BOLD fMRI. *Neuroimage* 26 1097–1108. 10.1016/j.neuroimage.2005.03.030 15886020

[B75] Robbins T. W. Gillan C. M. Smith D. G. de Wit S. Ersche K. D. (2012). Neurocognitive endophenotypes of impulsivity and compulsivity: Towards dimensional psychiatry. *Trends Cogn. Sci.* 16 81–91. 10.1016/j.tics.2011.11.009 22155014

[B76] Rogers T. D. Dickson P. E. Heck D. H. Goldowitz D. Mittleman G. Blaha C. D. (2011). Connecting the dots of the cerebro-cerebellar role in cognitive function: Neuronal pathways for cerebellar modulation of dopamine release in the prefrontal cortex. *Synapse* 65 1204–1212. 10.1002/syn.20960 21638338 PMC3854794

[B77] Rondi-Reig L. Paradis A. L. Fallahnezhad M. (2022). A liaison brought to light: Cerebellum-hippocampus, partners for spatial cognition. *Cerebellum* 21 826–837. 10.1007/s12311-022-01422-3 35752720

[B78] Sacchetti B. Baldi E. Lorenzini C. A. Bucherelli C. (2002). Cerebellar role in fear-conditioning consolidation. *Proc. Natl. Acad. Sci. U.S.A.* 99 8406–8411. 12034877 10.1073/pnas.112660399 PMC123080

[B79] Sacchetti B. Sacco T. Strata P. (2007). Reversible inactivation of amygdala, cerebellum, but not perirhinal cortex, impairs reactivated fear memories. *Eur. J. Neurosci.* 25 2875–2884. 17466022 10.1111/j.1460-9568.2007.05508.x

[B102] Schindelin J. Arganda-Carreras I. Frise E. Kaynig V. Longair M. Pietzsch T. (2012). Fiji: An open-source platform for biological-image analysis. *Nat. Methods* 9, 676–682. 10.1038/nmeth.2019 22743772 PMC3855844

[B80] Schmahmann J. D. Sherman J. C. (1998). The cerebellar cognitive affective syndrome. *Brain* 121(Pt 4) 561–579. 10.1093/brain/121.4.561 9577385

[B81] Schneider F. Habel U. Wagner M. Franke P. Salloum J. B. Shah N. J. (2001). Subcortical correlates of craving in recently abstinent alcoholic patients. *Am. J. Psychiatry* 158 1075–1083. 10.1176/appi.ajp.158.7.1075 11431229

[B82] Sha Z. Edmiston E. K. Versace A. Fournier J. C. Graur S. Greenberg T. (2020). Functional disruption of cerebello-thalamo-cortical networks in obsessive- compulsive disorder. *Biol. Psychiatry Cogn. Neurosci. Neuroimaging* 5 438–447. 10.1016/j.bpsc.2019.12.002 32033923 PMC7150632

[B83] Shen Z. Huang P. Wang C. Qian W. Yang Y. Zhang M. (2017). Increased network centrality as markers of relapse risk in nicotine-dependent individuals treated with varenicline. *Prog. Neuropsychopharmacol. Biol. Psychiatry* 75 142–147. 10.1016/j.pnpbp.2017.02.002 28185963

[B84] Slaker M. Churchill L. Todd R. P. Blacktop J. M. Zuloaga D. G. Raber J. (2015). Removal of perineuronal nets in the medial prefrontal cortex impairs the acquisition and reconsolidation of a cocaine-induced conditioned place preference memory. *J. Neurosci.* 35 4190–4202. 10.1523/JNEUROSCI.3592-14.2015 25762666 PMC4355195

[B85] Snider R. S. Maiti A. Snider S. R. (1976). Cerebellar pathways to ventral midbrain and nigra. *Exp. Neurol.* 53 714–728. 10.1016/0014-4886(76)90150-3 1001395

[B86] Sorg B. A. Berretta S. Blacktop J. M. Fawcett J. W. Kitagawa H. Kwok J. C. (2016). Casting a wide net: Role of perineuronal nets in neural plasticity. *J. Neurosci.* 36 11459–11468. 10.1523/JNEUROSCI.2351-16.2016 27911749 PMC5125213

[B87] Steinmetz J. E. Lavond D. G. Thompson R. F. (1989). Classical conditioning in rabbits using pontine nucleus stimulation as a conditioned stimulus and inferior olive stimulation as an unconditioned stimulus. *Synapse* 3 225–233. 10.1002/syn.890030308 2718098

[B88] Supple W. F. Leaton R. N. (1990). Lesions of the cerebellar vermis and cerebellar hemispheres: Effects on heart rate conditioning in rats. *Behav. Neurosci.* 104 934–947. 10.1037//0735-7044.104.6.934 2285492

[B89] Tomasi D. Wang G. J. Wang R. Caparelli E. C. Logan J. Volkow N. D. (2015). Overlapping patterns of brain activation to food and cocaine cues in cocaine abusers: Association to striatal D2/D3 receptors. *Hum. Brain Mapp.* 36 120–136. 10.1002/hbm.22617 25142207 PMC4306601

[B90] Tzschentke T. M. (2007). Measuring reward with the conditioned place preference (CPP) paradigm: Update of the last decade. *Addict. Biol.* 12 227–462. 10.1111/j.1369-1600.2007.00070.x 17678505

[B91] Utz A. Thürling M. Ernst T. M. Hermann A. Stark R. Wolf O. T. (2015). Cerebellar vermis contributes to the extinction of conditioned fear. *Neurosci. Lett.* 604 173–177. 10.1016/j.neulet.2015.07.026 26219987

[B92] Volkow N. D. Wang G.-J. Ma Y. Fowler J. S. Zhu W. Maynard L. (2003). Expectation enhances the regional brain metabolic and the reinforcing effects of stimulants in cocaine abusers. *J. Neurosci.* 23 11461–11468. 14673011 10.1523/JNEUROSCI.23-36-11461.2003 PMC6740524

[B93] Wagner M. J. Kim T. H. Kadmon J. Nguyen N. D. Ganguli S. Schnitzer M. J. (2019). Shared cortex-cerebellum dynamics in the execution and learning of a motor task. *Cell* 177 669–682.e24. 10.1016/j.cell.2019.02.019 30929904 PMC6500577

[B94] Wagner M. J. Kim T. H. Savall J. Schnitzer M. J. Luo L. (2017). Cerebellar granule cells encode the expectation of reward. *Nature* 544 96–100. 10.1038/nature21726 28321129 PMC5532014

[B95] Wang G.-J. Volkow N. D. Fowler J. S. Cervany P. Hitzemann R. J. Pappas N. R. (1999). Regional brain metabolic activation during craving elicited by recall of previous drug experiences. *Life Sci.* 64 775–784. 10.1016/s0024-3205(98)00619-5 10075110

[B96] Wang S. S.-H. Kloth A. D. Badura A. (2014). The cerebellum, sensitive periods, and autism. *Neuron* 83 518–532. 10.1016/j.neuron.2014.07.016 25102558 PMC4135479

[B97] Watabe-Uchida M. Zhu L. S. Ogawa S. K. Vamanrao A. Uchida N. (2012). Whole-brain mapping of direct inputs to midbrain dopamine neurons. *Neuron* 74 858–873. 10.1016/j.neuron.2012.03.017 22681690

[B98] Watson T. C. Becker N. Apps R. Jones M. W. (2014). Back to front: Cerebellar connections and interactions with the prefrontal cortex. *Front. Syst. Neurosci.* 8:4. 10.3389/fnsys.2014.00004 24550789 PMC3912388

[B99] Wei X. Li W. Chen J. Li Y. Zhu J. Shi H. (2020). Assessing drug cue-induced brain response in heroin dependents treated by methadone maintenance and protracted abstinence measures. *Brain Imaging Behav.* 14 1221–1229. 10.1007/s11682-019-00051-5 30734203

[B100] Winstanley C. A. Olausson P. Taylor J. R. Jentsch J. D. (2010). Insight into the relationship between impulsivity and substance abuse from studies using animal models. *Alcohol. Clin. Exp. Res.* 34 1306–1318. 10.1111/j.1530-0277.2010.01215.x 20491734 PMC3380443

[B101] Yoshida J. Oñate M. Khatami L. Vera J. Nadim F. Khodakhah K. (2022). Cerebellar contributions to the basal ganglia influence motor coordination, reward processing, and movement vigor. *J. Neurosci.* 42 8406–8415. 10.1523/JNEUROSCI.1535-22.2022 36351826 PMC9665921

